# Determination of nutritional sufficiency ranges for pomelo (*Citrus grandis* Osbeck) grown on alluvial soils using DRIS

**DOI:** 10.1371/journal.pone.0312231

**Published:** 2024-10-16

**Authors:** Nguyen Kim Quyen, Le Van Dang, Ngo Phuong Ngoc, Pham Thi Phuong Thao, Ngo Ngoc Hung

**Affiliations:** 1 Faculty of Agriculture and Fishery, University of Cuu Long, Vinh Long Province, Vietnam; 2 College of Agriculture, Can Tho University, Can Tho City, Vietnam; 3 United Graduate School of Agricultural Science, Tokyo University of Agriculture and Technology, Tokyo, Japan; Universiti Brunei Darussalam, BRUNEI DARUSSALAM

## Abstract

Pomelo is an important tropical fruit with a high nutrient content and economic value in the Vietnamese Mekong Delta (VMD). The Diagnosis and Recommendation Integrated System (DRIS) helps determine the leaf nutrient status of various plants worldwide. However, the DRIS-based nutritional balance in pomelo leaves remains to be established. Therefore, in this study, we aimed to (i) construct the DRIS norms and indices for nutrients, including macronutrients (N, P, K, Ca, and Mg) and trace elements (Cu, Fe, Zn, and Mn) in pomelo leaves, and (ii) establish nutrient sufficiency value ranges for sustainable pomelo cultivation in the VMD. We collected 270 leaf samples at three stages of pomelo growth, i.e., flowering, fruit development, and postharvest, and calculated DRIS indices for various nutrients. The DRIS indices established for various nutrients in pomelo leaves were accurate and reliable, as indicated by the high coefficient of determination (R^2^ = 0.43–0.93, p < 0.05) between nutrient concentrations and their DRIS indices. We observed that pomelo leaves were deficient in N (IN = −6.82), P (IP = −24.0), and Fe (IFe = −0.40) at the flowering stage and most deficient in P (IP = −15.6), K (IK = −11.7), Fe (IFe = −0.50), and Mn (IMn = −2.31) at the fruit development stage. However, only N (IN = −2.64) and P (IP = −13.4) shortages were observed at the postharvest stage. Thus, in this study, we evaluated nutrient value ranges (deficient, balanced, and excess) in pomelo leaves at their different growth stages and established DRIS indices for various nutrients. The results contribute to our understanding of the nutritional status of pomelo leaves, which can help growers improve plant health for sustainable pomelo production.

## Introduction

Pomelo (*Citrus grandis* Osbeck) is a citrus fruit cultivated in Southeast Asia where is tropical and subtropical climatic regions [1]. In Vietnam, various varieties of pomelo are grown from north to south. The ‘Nam Roi’ cultivar widely used in the VMD, and they are delicious, and contains few seeds and many essential nutrients [2]. The mean weight of these fruits’ ranges from 1.5–2.5 kg, and their skin transforms from green to yellow upon ripening. Pomelo is considered to be a poverty-reducing plant because of its high value in domestic and export markets [3]. Most pomelo cultivation areas in the VMD have alluvial soils with high fertility and nutrient abundance [4]. However, the production of pomelo has become unstable because of soil fertility reduction and poor nutrients management. Growers use large amounts of chemical fertilizers to obtain maximum yield. Past studies indicated that soil pH in pomelo orchards is significantly reduced because of the runoff/washing of exchangeable cations from surface soil under tropical conditions [4, 5], which may negatively affect the availability of soil nutrients such as P, Ca, and Mg, resulting in reduced nutrient uptake in plants.

Mineral nutrients play crucial roles in citrus growth and yield [6]. N regulates in many biochemical and physiological characteristics in citrus plants, including root activity and development, photosynthesis, and crop productivity [7]. It has been shown that P is required for many vital functions in plants [8]. Moreover, P is required for optimal citrus blossom, fruit setting and development [9]. K is an essential element in citrus juices and plays important roles in the regulation of water relations, and acid metabolism [10]. Ca plays a crucial role in forming pedicel attachment to the proximal fruit, thereby preventing fruit drop [11]. Astiari et al. [12] reported that Ca deficiency produces rough, thick, and poor-quality rind. Mg is vital for chlorophyll and sugar production. Cu helps decrease fruit drop rate and improves fruit quality [13]. Fe enhances the contents of Brix, total sugar, and juices in fruits [14]. Zn is considered the main factor that increases the ascorbic acid and anthocyanin content in the fruit [15]. Mn decreases the acidity of fruit juices [16]. Xu et al. [17] reported an interaction between the mineral nutrients within plants. For example, a reduction in Mg uptake occurs at high K concentrations in crops, whereas the absorption of Na and Ca increases with K deficiency [17]. Therefore, the determination and balancing of mineral nutrients in plants is important for maintaining crop health and sustainable agriculture.

Several studies [18, 19] have described methods for evaluating the nutritional status of plants through leaf analysis. The Critical Value Approach (CVA) evaluates leaf concentrations of nutrients at above 90% of maximum productivity [20]. This method would be effective if only a nutrient showed deficient results because the relationships between nutrients were not assessed [21]. The Sufficiency Range Approach (SRA) is used to evaluate the status of nutrients in leaves, including deficiency or excess [22]. However, it does not evaluate balance or interactions of nutrients [23]. Thus, the disadvantages of the CVA and SRA methods are that they cannot be used to evaluate the interactions between mineral nutrients or calculate the critical levels of each nutrient [24]. Occasionally, the results contain more errors when applied to different plant growth stages [25]. The Diagnostic and Recommendation Integrated System (DRIS) was developed by Beaufils [26] and has since been improved or modified by many crop scientists for easier and more accurate use [27, 28]. There are two main steps for establishing a DRIS: (1) determining DRIS norms based on nutrient ratio pairs and (2) calculating DRIS indices based on DRIS norms [29]. The DRIS indices indicate whether leaves are deficient (negative value), balanced (zero value), or have an excess (positive value) of mineral nutrients. The benefit of using the DRIS is the determination of specific nutrients based on the values of all nutrients in the DRIS norms. Therefore, these results are reliable and precise for determining the nutritional status of crops [30, 31]. Globally, the DRIS has been applied to determine the sufficiency ranges for mangoes [32], guavas [33], star apples [24], and pineapples [31]. DRIS is an interesting method for assessing the nutritional status of crops; however, DRIS norms need to be established for each crop. This is because the accuracy of DRIS is affected by environmental conditions (e.g., soil and climate) and plant species.

DRIS has been used to assess leaf nutritional status in star apples [24], durians [34], and pineapples [31]. However, information on the application of DRIS to pomelos is lacking. Therefore, to establish DRIS norms and indices for pomelos, we investigated 90 pomelo orchards in the VMD and examined soil properties and concentrations of leaf mineral nutrients (N−P−K−Ca−Mg−Cu−Fe−Zn−Mn). The objectives of the study were to (i) construct and create a DRIS for pomelo leaves at three stages (flowering, fruit development, and postharvest) and (ii) calculate and recommend optimal nutrient concentration ranges for pomelo leaves.

## Materials and methods

### Study area and environmental conditions

‘Nam Roi’ pomelo is cultivated in the Hau Giang and Vinh Long provinces, VMD [35]. The Chau Thanh district of Hau Giang province is considered the largest area of pomelo production [3]. Therefore, we investigated the leaf mineral nutrients and soil characteristics of 90 pomelo gardens in this region. The People’s Committee of Hau Giang approved and supported field surveys, contract number 14/HĐ-KHCN. The meteorological characteristics of this area have been described in our previous study [5]; the average monthly rainfall and air temperatures between January and December 2018 were 160 mm and 27.2°C, respectively.

In this study, most pomelo trees were grown on raised bed soil with a distance of 4.0 m × 4.0 m [36]. Our previous study [37] showed that the average amounts of N−P−K fertilizers applied for pomelo were 652–164–149 g tree^−1^ year^−1^, respectively. They were separated into six different application times [5]: (1) the application of 20%N–30%P after a month of fruit harvesting; (2) 15%N–40%P–30%K 2 months before pomelo blossoming; (3) 20%N–10%P–15%K after a month of fruit setting; (4) 25%N− 10%P–15%K after 2.5 months of fruit setting; (5) 20%N–10%P–20%K after 4 months of fruit setting; and (6) 20%K 2 months before fruit harvesting. Complex fertilization schedules, such as N–P–K + trace elements (TEs) (e.g., 16–16–16+TEs or 20–20–15+TEs) and diammonium phosphate (DAP, 18%N–46%P) were used by farmers. However, the amounts of TEs in fertilizers remain unknown. Therefore, we considered and recorded pomelo orchards wherein TEs (Cu, Fe, Zn, and Mn) were applied.

### Sampling, collection, and analysis

#### Plants

In this study, 90 pomelo orchards were surveyed through 2018. The ages of the pomelo trees in this study ranged from 4.0 to 4.5 years, and they have been cultivated since 2014. We collected leaves at three different pomelo growth stages: flowering (30–50% of flowers opened, May 2018), fruit development (2 months after fruit set, August 2018), and postharvest (1 month after fruit harvest, December 2018). At each stage, we collected the third and fourth pomelo leaves from the 3-month-old branches of four plants per orchard, and there were 16 leaves from each orchard. The leaves did not exhibit any symptoms of disease or pest damage. A total of 270 leaf samples were taken (90 samples in three stages). After taking, the leaves were washed at least three times with distilled water. Finally, they were put in an oven at 70 °C for 80 h, then ground to a powder, and stored in a plastic bag to analyze their mineral nutrient contents.

The leaf mineral nutrient contents were measured as described by Houba et al. [38]. First, the leaf samples were digested to transform them from their organic state to an inorganic state using a solution containing 36 mL water, 12 g salicylic acid, and 200 mL 96% H_2_SO_4_. During the digestion stage, we added 3–5 drops of 30% H_2_O_2_ solution. After leaf sample digestion, N concentration was measured using the Kjeldahl method. Samples were distilled with 20 mL NaOH (40%) and titrated with 0.01 N H_2_SO_4_ solution. Total P was extracted using a solution containing H_2_SO_4_ (96%) and HClO_4_ and colorimetric ascorbic acid solution and measured using an ultraviolet (UV) spectrophotometer (UV-1800, Shimadzu), and K, Ca, Mg, Cu, Fe, Zn, and Mn were determined using atomic absorption spectrophotometry (AAS, iCE 3500, Thermo Scientific) at the wavelengths of 766.5, 422.7, 285.2, 342.8, 248.3, 213.9, and 279.5 nm, respectively.

#### Soils

Soil samples were collected from 90 pomelo orchards during the first stage of leaf collection (flowering) in May 2018. All soil samples (0–20 cm) were air-dried for 15 d and then ground to pass through 0.5- and 2-mm sieves. Soil properties, such as pH, electrical conductivity (EC), soil organic carbon (SOC), total N (N_tot_), available P (P_avail_), and the concentrations of mineral nutrients (Cu, Fe, Zn, and Mn) and exchangeable cations (K^+^, Ca^2+^, and Mg^2+^), were measured. The soil analysis was conducted as described by Houba et al. [38]. Briefly, soil pH and EC were determined at a 1:5 soil: water ratio (10 g soil: 50 mL distilled water) and measured using a digital pH/EC meter. The soil organic matter content was measured using the Walkley–Black method, and soil was extracted using a solution containing 10 mL K_2_Cr_2_O_7_ + 20 mL H_2_SO_4_ (96%) and titrated with a 0.5 M FeSO_4_ solution. N_tot_ was determined using the Kjeldahl method, i.e., soil was extracted using a solution containing H_2_SO_4_ (96%)−CuSO_4_−Se at a ratio of 100–10–1 and distilled with 10 mL NaOH (40%) and then titrated with 0.01 N H_2_SO_4_ solution. P_avail_ was analyzed using the Bray II method, i.e., soil was extracted using a solution containing 0.1 N HCl + 0.03 N NH_4_F at a ratio of 1:7, and the P content in the extract was measured using a UV spectrophotometer (UV-1800; Shimadzu). Exchangeable cations (K^+^, Ca^2+^, and Mg^2+^) were extracted using 0.1 M BaCl_2_. Fe present in the soil was extracted using oxalate/ oxalic acid. Cu, Mn, and Zn were extracted using nitric/ perchloric acid. Finally, the concentrations of K^+^, Ca^2+^, Mg^2+^, Fe, Mn, Cu, and Zn were measured using an AAS, as described above.

### DRIS norms

To establish the DRIS norms for pomelos, the data were first divided into two (low and high) yield groups. According to Aliyu et al. [39], the yield groups were calculated based on the average of all pomelo fruit yield populations + (0.5 × standard deviation). We recorded pomelo fruit yields from the 90 orchards in November 2018. We successfully separated two yield groups, including the high-fruit yield (≥14.7 t ha^−1^) and low-fruit yield (<14.7 t ha^−1^) groups (Table 1). Finally, DRIS norms were established as follows: (i) the construction of nutrient ratio pairs (A/B, B/A, B/C, C/B, … Y/Z, Z/Y) and (ii) the determination of mean values, coefficient of variation (CV), and variance (σ^2^) of the high- and low-yield groups.

**Table 1:** Fruit yields of the low- and high-yield pomelo groups for diagnosis and recommendation integrated system norms.

Parameters	Min	Mean	Max	SD	CV (%)
Total fruit yield (n = 90)	4.77	12.5	20.2	4.37	34.8
LYG (n = 50)	4.77	9.15	13.3	2.51	27.5
HYG (n = 40)	14.7	16.8	20.2	1.56	9.29

LYG: low fruit yield group; HYG: high fruit yield group; SD: standard deviation; CV: coefficient of variation.

### Calculation of DRIS indices

The DRIS indices were calculated after establishing the DRIS norms. According to Khuong et al. [25] and Aliyu et al. [39], the DRIS indices need to be determined using the following equations:

$$\text{IA}=\left(\text{f}\left(\text{A}/\text{B}\right)+\text{f}\left(\text{A}/\text{C}\right)+\ldots +\text{f}\left(\text{A}/\text{Z}\right)\right)/\text{X}$$ (1)

$$\text{IB}=\left(-\text{f}\left(\text{A}/\text{B}\right)+\text{f}\left(\text{A}/\text{C}\right)+\ldots +\text{f}\left(\text{A}/\text{Z}\right)\right)/\text{X}$$ (2)

where IA and IB are the DRIS indices for nutrients A and B, respectively. f(A/B) is the function calculated for the A and B nutrient ratio; f(A/B) = [((A/B)/(a/b)) − 1] × (1000/CV) if (A/B) ≥ (a/b), and f(A/B) = [1 − ((a/b)/(A/B))] × (1000/CV) if (A/B) < (a/b). A/B is the nutrient ratio for diagnosis, and a/b is the value of the nutrient ratio determined from the DRIS norms. CV is the coefficient of variation, and X is the number of functions used for calculating the total nutrients.

### Establishment of optimum nutrient ranges in pomelo leaves

The ranges of optimum nutrients (ON) in leaves were determined using the mean (M) and SD of mineral nutrient concentrations in the high-yielding population [29]. Leaf nutrient ranges of pomelo leaves were computed as described by Morales et al. [29], as excessive [ON > (4/3 × SD + M)], high [(2/3 × SD + M) < ON < (M + SD × 4/3)], optimum [(**–** 2/3 × SD + M) < ON < (M + SD × 2/3)], low [(**−**4/3 × SD + M) < ON < (M + SD × −2/3)], and deficient (< −4/3 × SD + M). We established five classes of foliar pomelo diagnoses.

### Statistical analysis

Data was summarized using Microsoft Excel (ver. 16), and IBM SPSS Statistics (ver. 20) was used for data analysis. We used the Student’s *t*-test to compare the averages of soil properties between the low- and high-yield groups. Relationships between the soil parameters were assessed using Pearson’s correlation analysis.

## Results

### Soil characteristics of the study sites

Table 2 describes the soil properties of the two pomelo fruit yield groups. The soil pH in the low-yield group (LYG) was lower by 0.52 units than that in the high-yield group (HYG). The SOC content in the HYG was higher by 3.2 g C kg^−1^ than that in the LYG. Similarly, P_avail_ concentrations in the HYG were higher by 5.8 mg P kg^−1^ than in the LYG. Further, the results of the exchangeable Ca^2+^ content analysis were similar; Ca^2+^ occurred at 4.98 and 5.36 cmol kg^−1^ concentrations in the LYG and HYG, respectively. However, there were no significant differences in EC, N_tot_, K^+^, Mg^2+^, Cu, Fe, Zn, or Mn between the LYG and HYG. The value of EC ranged between 0.44 and 0.46 mS cm^−1^ in both yield groups. Similarly, the mean values of N_tot_ and K^+^ and Mg^2+^ ion concentrations were 1.40 g kg^−1^, 0.31 cmol kg^−1^, and 2.68 cmol kg^−1^, respectively, whereas the mean values of Cu, Fe, Zn, and Mn contents were 26.3, 11.1, 61.6, and 27.1 mg kg^−1^, respectively, in the entire population.

**Table 2:** Soil properties at 0–20 cm depth in the surveyed pomelo orchards.

Properties	Low yield group (n = 50)			High yield group (n = 40)			t-test
	Mean	CV (%)	SD	Mean	CV (%)	SD	
pH_H2O_	4.83^b^	12.5	0.60	5.35^a^	10.6	0.57	***
EC (mS cm^−1^)	0.46	19.3	0.09	0.44	20.4	0.09	ns
SOC (g C kg^−1^)	28.4^b^	15.2	4.32	31.6^a^	11.3	3.56	***
N_tot_ (g kg^−1^)	1.37	21.9	0.30	1.43	25.4	0.36	ns
P_avail_ (mg kg^−1^)	23.6^b^	28.8	6.79	29.4^a^	18.2	5.34	***
K^+^ (cmol kg^−1^)	0.31	27.4	0.08	0.30	31.0	0.09	ns
Ca^2+^ (cmol kg^−1^)	4.98^b^	12.3	0.61	5.36^a^	10.2	0.55	**
Mg^2+^ (cmol kg^−1^)	2.59	16.3	0.42	2.76	26.6	0.73	ns
Cu (mg kg^−1^)	26.0	12.0	3.12	26.6	10.7	2.85	ns
Fe (mg kg^−1^)	11.1	25.9	2.87	11.1	21.5	2.38	ns
Zn (mg kg^−1^)	61.8	11.4	7.08	61.4	11.1	6.79	ns
Mn (mg kg^−1^)	27.2	10.1	2.76	27.0	14.3	3.87	ns

EC: electrical capacity; SOC: soil organic carbon; N_tot_: total N; P_avail_: available P; ns: no significant;

** and *** indicates p < 0.01 and p < 0.001, respectively.

Low yield group <14.7 t ha^−1^ and high yield group ≥ 14.7 t ha^−1^; SD: standard deviation; CV: coefficient of variation.

**Fig 1:**
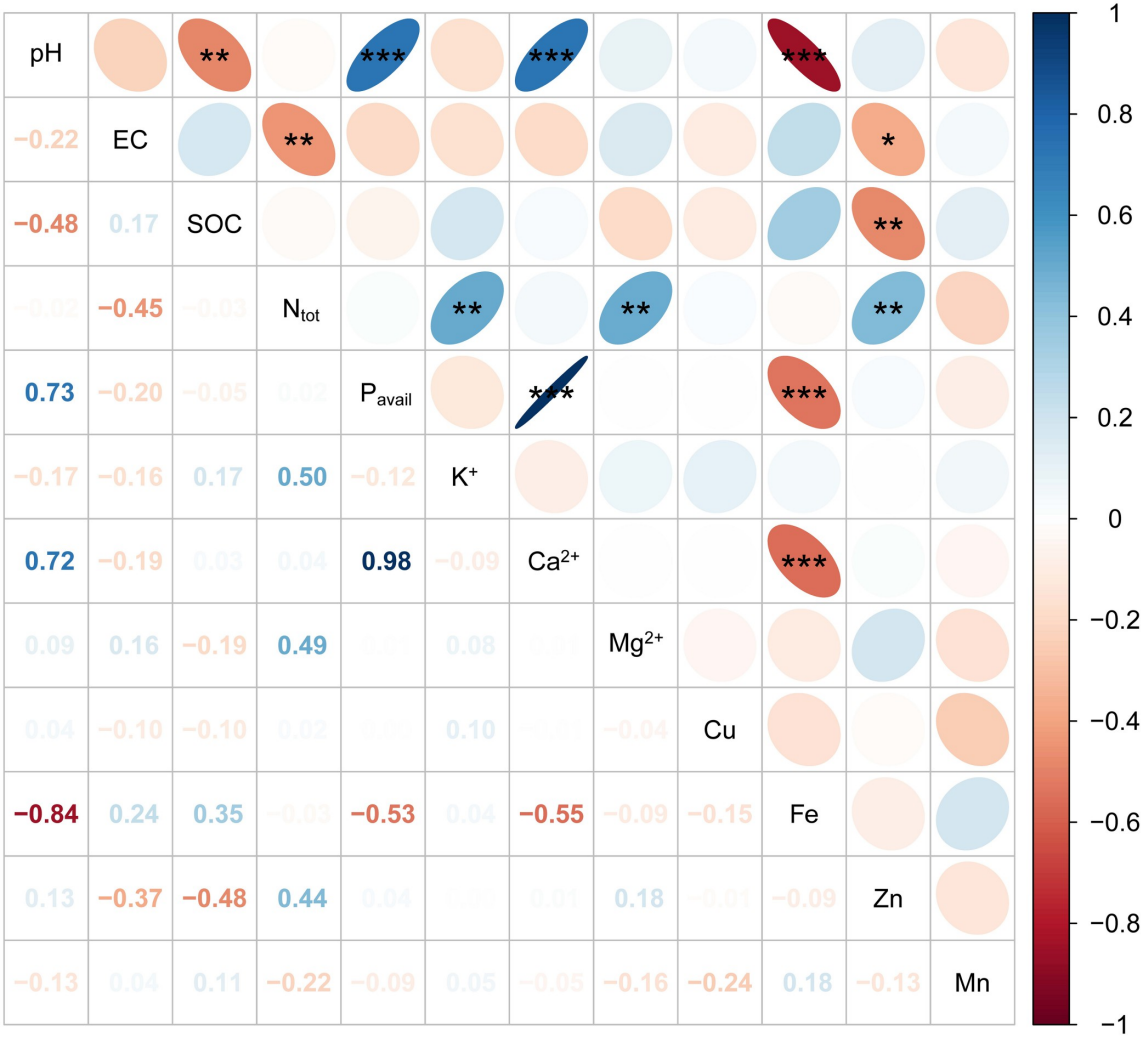
Pearson correlation coefficient (r) among soil properties in the low fruit yield. *, **, and *** indicates p < 0.05, p < 0.01, and p < 0.001, respectively.

**Fig 2:**
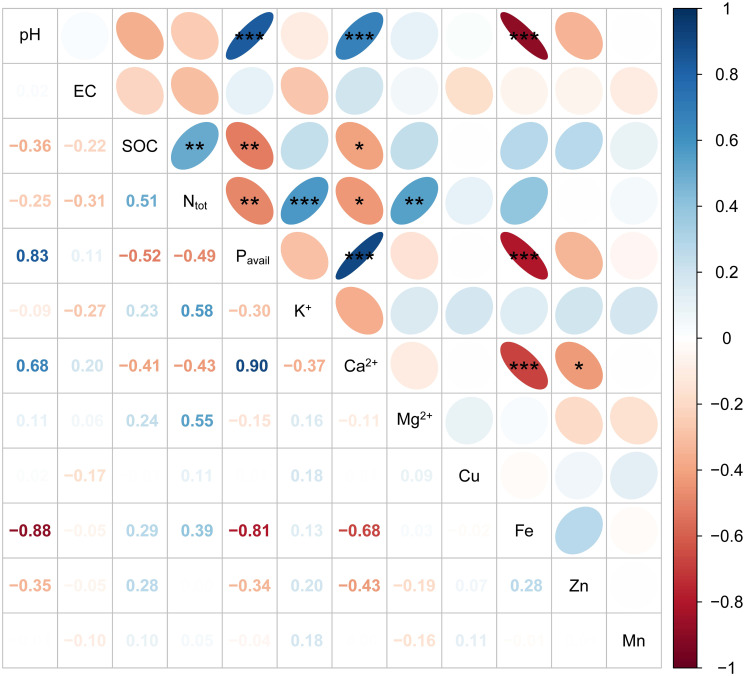
The correlation coefficient (Pearson) measures the relationship among soil properties in areas with high fruit yield. *, **, and *** indicates p < 0.05, p < 0.01, and p < 0.001, respectively.

The relationships between the soil parameters in LYG and HYG are shown in Figs 1 & 2. SOC in the LYG was positively correlated with pH (r = −0.48**) and Zn (r = −0.48**) (Fig 1). A positive correlation exists between pH and P_avail_ (r = 0.72***) and between pH and Ca^2+^ (r = 0.72***). Similarly, P_avail_ was positively correlated with pH (r = 0.73***) and Ca^2+^ (r = 0.98***) but was negatively correlated with Fe (r = −0.53***). Negative correlations were observed between Fe and pH (r = −0.84***) and between Fe and Ca^2+^ (r = −0.55***). A correlation was observed among the soil properties in the HYG (Fig 2). pH was positively correlated with P_avail_ (r = 0.83***) and Ca^2+^ (r = 0.68***). Similarly, a negative correlation was recorded between Fe and P_avail_ (r = −0.81***), Fe and pH (r = −0.88***), and Fe and Ca^2+^ (r = −0.68***) in the HYG soil.

### DRIS norms

Table 3 shows the values of the leaf nutritional components and nutrient pair ratios in the LYG and HYG. The means of macronutrients (N, P, K, Ca, and Mg) in the LYG were 24.9, 1.23, 19.9, 24.8, and 19.5 g kg^−1^, respectively. These values in the HYG were 27.8, 1.63, 20.8, 24.6, and 19.6 g kg^−1^. The concentrations of micronutrients (Cu, Fe, Zn, and Mn) in pomelo leaves of LYG were 27.3, 128, 31.1, and 39.5 mg kg^−1^, respectively. While they were 25.4, 142, 34.0, and 43.1 mg kg^−1^ in HYG, respectively. After establishing the nutrient ratios, we selected 36 that were used to calculate the DRIS indices. These included N/10P, N/K, N/10Ca, N/Mg, 10N/Cu, 100N/Fe, 10N/Zn, 10N/Mn, P/K, P/Ca, P/Mg, 10P/Cu, 100P/Fe, 10P/Zn, 100P/Mn, K/10Ca, K/Mg, K/Cu, 100K/Fe, 10K/Zn, 10K/Mn, Ca/Mg, 10Ca/Cu, 1000Ca/Fe, 100Ca/Zn, 100Ca/Mn, Mg/Cu, 100Mg/Fe, 10Mg/Zn, 100Mg/Mn, 100Cu/Fe, 10Cu/Zn, 10Cu/Mn, Fe/10Zn, Fe/10Mn, and Zn/Mn. The CV values for all nutrient ratios were approximately 30% lower in both the high- and low-fruit-yield group. Thus, DRIS norms were established to be highly reliable and accurate as the basis for DRIS indices.

**Table 3:** Contents of mineral nutrients in pomelo leaves and DRIS norms.

Parameters	Low yield group (n = 150)			High yield group (n = 120)		
	Mean	CV (%)	Variance (σ^2^)	Mean	CV (%)	Variance (σ^2^)
**Nutrients**						
N (g kg^−1^)	24.9	13.7	7.743	27.8	16.8	10.78
P (g kg^−1^)	1.23	18.5	0.052	1.63	11.4	0.034
K (g kg^−1^)	19.9	14.9	8.732	20.8	15.2	9.958
Ca (g kg^−1^)	24.8	16.1	15.97	24.6	15.1	13.74
Mg (g kg^−1^)	19.5	15.0	8.517	19.6	16.5	10.35
Cu (mg kg^−1^)	27.3	11.6	9.997	25.4	12.3	9.839
Fe (mg kg^−1^)	128	21.7	772.9	142	22.2	996.4
Zn (mg kg^−1^)	31.1	16.4	26.15	34.0	12.9	19.05
Mn (mg kg^−1^)	39.5	24.0	90.37	43.1	22.5	94.08
**DRIS norms establishment**						
N/10P	2.0724	13.60	0.0795	1.7201	8.83	0.0231
N/K	1.3844	133.0	3.3913	1.3665	14.69	0.0403
N/10Ca	0.1028	18.37	0.0004	0.1159	17.29	0.0004
N/Mg	1.3033	16.77	0.0478	1.4567	15.49	0.0509
10N/Cu	9.2324	14.16	1.7098	11.125	14.76	2.6954
100N/Fe	20.288	21.93	19.807	20.330	18.95	14.843
10N/Zn	8.1976	17.67	2.0997	8.3216	14.27	1.4108
10N/Mn	6.6356	24.68	2.6832	6.7291	19.70	1.7570
P/K	0.0691	151.1	0.0109	0.0802	18.02	0.0002
P/Ca	0.0507	24.09	0.0001	0.0681	21.37	0.0002
P/Mg	0.0643	23.46	0.0002	0.0851	16.28	0.0002
10P/Cu	0.4556	21.49	0.0096	0.6511	15.81	0.0106
100P/Fe	1.0004	26.68	0.0713	1.1926	20.55	0.0600
10P/Zn	0.4046	23.82	0.0093	0.4885	18.13	0.0078
100P/Mn	3.2657	28.66	0.8762	3.9570	23.45	0.8612
K/10Ca	0.0820	21.49	0.0003	0.0868	23.36	0.0004
K/Mg	1.0317	15.49	0.0255	1.0931	25.24	0.0761
K/Cu	0.7390	19.60	0.0210	0.8344	22.41	0.0350
100K/Fe	16.208	25.33	16.855	15.366	27.70	18.111
10K/Zn	6.5650	21.52	1.9975	6.2330	20.75	1.6725
10K/Mn	5.2644	24.77	1.7004	5.0182	23.40	1.3788
Ca/Mg	1.2980	20.85	0.0733	1.2968	23.92	0.0962
10Ca/Cu	9.1685	16.82	2.3794	9.8639	22.86	5.0858
1000Ca/Fe	202.42	26.11	2795.3	178.03	20.12	1282.4
100Ca/Zn	82.264	24.14	394.40	73.793	21.15	243.67
100Ca/Mn	66.146	29.16	372.26	59.061	21.95	168.06
Mg/Cu	0.7255	19.77	0.0206	0.7798	19.55	0.0232
**DRIS norms establishment**						
100Mg/Fe	15.875	25.22	16.032	14.324	25.01	12.832
10Mg/Zn	6.4261	21.08	1.8365	5.8643	22.08	1.6773
100Mg/Mn	51.720	25.15	169.22	47.563	26.83	162.89
100Cu/Fe	22.268	23.20	26.702	18.774	25.28	22.523
10Cu/Zn	9.0283	20.94	3.5754	7.6424	20.62	2.4838
10Cu/Mn	7.3033	27.53	4.0436	6.1618	23.27	2.0559
Fe/10Zn	0.4195	23.56	0.0098	0.4230	23.07	0.0095
Fe/10Mn	0.3363	25.85	0.0076	0.3404	25.97	0.0078
Zn/Mn	0.8242	25.56	0.0444	0.8230	23.94	0.0388

Low yield group <14.7 t ha^−1^ and high yield group ≥ 14.7 t ha^−1^; CV: coefficient of variation.

### Establishment of DRIS indices for pomelo

Table 4 shows the nutrient indices determined for pomelo at the three different growth stages. N, P, and Fe contents were below normal limits in pomelo leaves at the flowering stage, with indices below zero. The N and P indices with values of −6.82 and −24.0, respectively, indicated an extreme deficiency of these nutrients in pomelo leaves, whereas these leaves were slightly deficient in Fe, with a Fe index of −0.40. In contrast, the contents of K, Ca, Mg, Cu, Zn, and Mn in pomelo leaves were high because the indices were above zero. The nutrients P, K, Fe, and Mn are considered limiting factors at the fruit developmental stage. Of the nine nutrients investigated postharvest, only the indices of N and P were less than zero.

**Table 4:** Nutrient indices established for pomelo leaves.

Nutrients	Nutrient indices at the different growth stages		
	Flowering (n = 90)	Fruit development (n = 90)	Postharvest (n = 90)
N	−6.82	2.63	−2.64
P	−24.0	−15.6	−13.4
K	4.86	−11.7	0.59
Ca	4.43	8.78	2.75
Mg	7.94	3.71	2.95
Cu	10.4	14.7	5.21
Fe	−0.40	−0.50	0.76
Zn	0.15	0.31	1.77
Mn	3.79	−2.31	2.05

**Fig 3:**
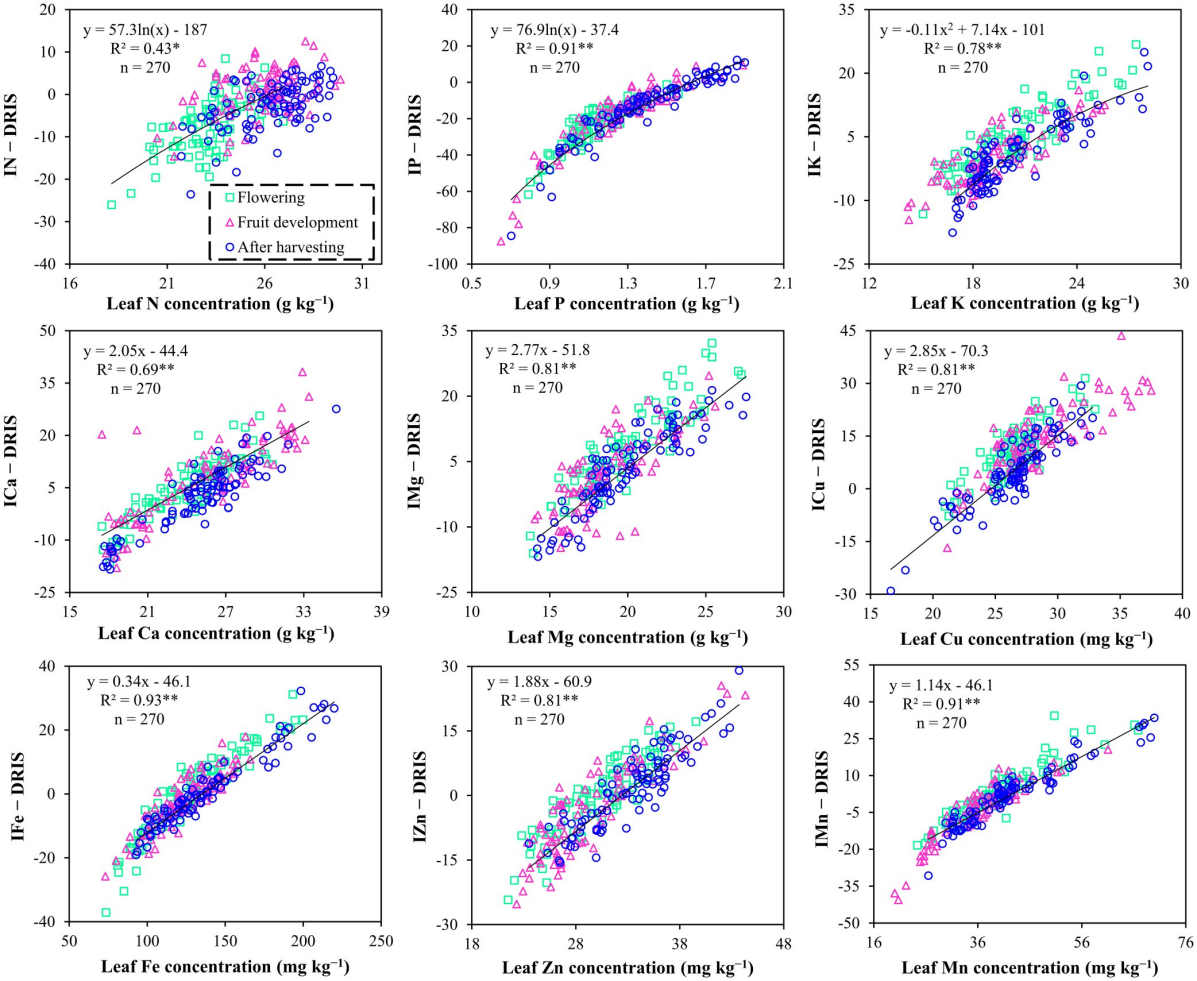
The relationship between the leaf mineral nutrient contents and their corresponding nutrients DRIS indices. (IN: N index… IMn: Mn index); p < 0.05 (*) and p < 0.01 (**).

Fig 3 provides the relationship between leaf nutrient (N, P, K, Ca, Mg, Cu, Fe, Zn, and Mn) content and the DRIS indices, which were determined by regression analysis. The results showed a strong positive relationship between nutrient concentration and the DRIS index in all cases. Therefore, highly reliable DRIS indices were established for the nutrient diagnoses of pomelo trees. In this study, the DRIS indices of microelements (Cu, Fe, Zn, and Mn) aligned relatively more to their contents in leaves compared with that for the DRIS indices of macronutrients (N, P, K, Ca, and Mg), as the values of *R*^*2*^ between the concentrations and DRIS indices of micronutrients exceeded 0.80.

### Concentrations of mineral nutrients in pomelo leaves at different growth stages

Table 5 shows the effects of the growth stages of pomelo on leaf nutrient concentrations. Mineral nutrient content was higher at the postharvest stage than at the flowering and fruit development stages, except for Cu content. The concentrations of N, P, Fe, and Zn during the post-harvest stage at 3.3 g N kg^−1^, 0.26 g P kg^−1^, 11.0 mg Fe kg^−1^, and 1.7 mg Zn kg^−1^ were higher than those during the flowering stage. Moreover, the contents of K, Mg, Zn, and Mn during the postharvest stage at 1.3 g K kg^−1^, 1.0 g Mg kg^−1^, 3.1 mg Zn kg^−1^, and 5.5 mg Mn kg^−1^ were higher than those during the fruit development stage. Therefore, nutrients were most abundant in pomelo leaves at the postharvest stage.

**Table 5:** Comparison of mineral nutrients in pomelo leaf under different development stages.

Nutrients	Flowering (n = 90)		Fruit development (n = 90)		Postharvest (n = 90)		*Tukey test*
	Mean	Standard deviation	Mean	Standard deviation	Mean	Standard deviation	
N (g kg^−1^)	23.3^b^	1.72	25.9^a^	1.88	26.3^a^	1.99	***
P (g kg^−1^)	1.14^c^	0.16	1.27^b^	0.23	1.40^a^	0.28	***
K (g kg^−1^)	20.3^a^	2.72	19.2^b^	3.25	20.5^a^	2.81	**
Ca (g kg^−1^)	23.9^b^	3.36	25.4^a^	4.70	25.1^ab^	3.58	*
Mg (g kg^−1^)	19.9^a^	3.02	18.8^b^	2.61	19.8^a^	3.08	*
Cu (mg kg^−1^)	26.5^b^	2.24	28.3^a^	3.80	26.5^b^	3.07	***
Fe (mg kg^−1^)	127^b^	29.8	124^b^	20.0	138^a^	32.4	**
Zn (mg kg^−1^)	30.4^b^	4.02	30.3^b^	6.16	33.4^a^	4.32	***
Mn (mg kg^−1^)	40.6^a^	9.24	36.8^b^	8.67	42.3^a^	10.0	**

Different letters in each row indicate significant differences at p < 0.05 (*), p < 0.01 (**), and p < 0.001 (***).

### Optimal nutrient ranges for pomelo leaves during the different growth stages

We determined the optimal nutrient ranges for pomelo leaves during the different developmental stages based on the nutrient concentrations in the HYG (Table 6). In this study, we divided pomelo plants into five classes for leaf nutrient diagnosis: deficient, low content, optimum content, high content, and in excess for each nutrient during the flowering, fruit development, and postharvest stages.

**Table 6:** Optimal nutrient ranges for pomelo leaves during the different growth stages.

Growth stage	Nutrient	In excess	High content	Optimum content	Low content	Deficient
**Flowering**	N (g kg^−1^)	> 29.3	28.3–29.3	26.0–28.2	24.9–25.9	< 24.8
	P (g kg^−1^)	> 1.79	1.68–1.79	1.42–1.67	1.30–1.41	< 1.29
	K (g kg^−1^)	> 25.4	23.2–25.4	18.8–23.1	16.5–18.7	< 16.4
	Ca (g kg^−1^)	> 28.8	26.3–28.8	21.0–26.2	18.4–20.9	< 18.3
	Mg (g kg^−1^)	> 23.7	21.8–23.7	17.6–21.7	15.6–17.5	< 15.5
	Cu (mg kg^−1^)	> 29.1	27.4–29.1	23.8–27.3	22.1–23.7	< 22.0
	Fe (mg kg^−1^)	> 185	163.1–185	120.1–163	98.1–120	< 98.0
	Zn (mg kg^−1^)	> 40.9	38.6–40.9	33.7–38.5	31.3–33.6	< 31.2
	Mn (mg kg^−1^)	> 46.0	42.6–46.0	35.5–42.5	32.0–35.4	< 31.9
**Fruit development**	N (g kg^−1^)	> 29.3	28.8–29.3	27.6–28.7	27.0–27.5	< 26.9
	P (g kg^−1^)	> 1.88	1.76–1.88	1.50–1.75	1.37–1.49	< 1.36
	K (g kg^−1^)	> 25.9	23.5–25.9	18.5–23.4	16.1–18.4	< 16.0
	Ca (g kg^−1^)	> 28.2	27.2–28.2	25.0–27.1	23.9–24.9	< 23.8
	Mg (g kg^−1^)	> 23.7	21.3–23.7	16.4–21.2	13.9–16.3	< 13.8
	Cu (mg kg^−1^)	> 29.5	27.1–29.5	22.1–27.0	19.6–22.0	< 19.5
	Fe (mg kg^−1^)	> 201	176.1–201	127.1–176	103.1–127	< 103
	Zn (mg kg^−1^)	> 37.0	34.7–37.0	30.0–34.6	27.6–29.9	< 27.5
	Mn (mg kg^−1^)	> 67.2	59.1–67.2	42.7–59.0	34.5–42.6	< 34.4
**Postharvest**	N (g kg^−1^)	> 29.6	29.1–29.6	28.0–29.0	27.4–27.9	< 27.3
	P (g kg^−1^)	> 1.89	1.83–1.89	1.70–1.82	1.63–1.69	< 1.62
	K (g kg^−1^)	> 23.8	22.2–23.8	18.9–22.1	17.2–18.8	< 17.1
	Ca (g kg^−1^)	> 30.7	27.6–30.7	21.1–27.5	17.9–21.0	< 17.8
	Mg (g kg^−1^)	> 24.3	22.4–24.3	18.5–22.3	16.5–18.4	< 16.4
	Cu (mg kg^−1^)	> 30.4	28.4–30.4	24.4–28.3	22.3–24.3	< 22.2
	Fe (mg kg^−1^)	> 165	149.1–165	117.1–149	101.1–117	< 101
	Zn (mg kg^−1^)	> 40.4	37.0–40.4	29.8–36.9	26.3–29.7	< 26.2
	Mn (mg kg^−1^)	> 46.2	43.0–46.2	36.3–42.9	32.9–36.2	< 32.8

In the flowering stage, the concentration of nutrients (N, P, K, Ca, Mg, Cu, Fe, Zn, and Mn) was considered as a deficient if they were less than 24.8 g kg^−1^, 1.29 g kg^−1^, 16.4 g kg^−1^, 18.3 g kg^−1^, 15.5 g kg^−1^, 22.0 mg kg^−1^, 98.0 mg kg^−1^, 31.2 mg kg^−1^, and 31.9 mg kg^−1^, respectively; the optimal ranges were 26.0–28.2 g N kg^−1^, 1.42–1.67 g P kg^−1^, 18.8–23.1 g K kg^−1^, 21.0–26.2 g Ca kg^−1^, 17.6–21.7 g Mg kg^−1^, 23.8–27.3 mg Cu kg^−1^, 120–163 mg Fe kg^−1^, 33.7–38.5 mg Zn kg^−1^, and 35.5–42.5 mg Mn kg^−1^, respectively; and these nutrients were considered excessive when they were higher than 29.3 g N kg^−1^, 1.79 g P kg^−1^, 25.4 g K kg^−1^, 28.8 g Ca kg^−1^, 23.7 g Mg kg^−1^, 29.1 mg Cu kg^−1^, 185 mg Fe kg^−1^, 40.9 mg Zn kg^−1^, and 46.0 mg Mn kg^−1^, respectively.

For the fruit development stage, the contents of leaf nutrients (N, P, K, Ca, Mg, Cu, Fe, Zn, and Mn) ranged from 27.6–28.7 g N kg^−1^, 1.50–1.75 g P kg^−1^, 18.5–23.4 g K kg^−1^, 25.0–27.1 g Ca kg^−1^, 16.4–21.2 g Mg kg^−1^, 22.1–27.0 mg Cu kg^−1^, 127.1–176 mg Fe kg^−1^, 30.0–34.6 mg Zn kg^−1^, and 42.7–59.0 mg Mn kg^−1^, respectively, and were estimated as optimal for pomelo.

Finally, the optimal nutrient ranges (N, P, K, Ca, Mg, Cu, Fe, Zn, and Mn) for pomelo leaf postharvest were 28.0–29.0 g N kg^−1^, 1.70–1.82 g P kg^−1^, 18.9–22.1 g K kg^−1^, 21.1–27.5 g Ca kg^−1^, 18.5–22.3 g Mg kg^−1^, 24.4–28.3 mg Cu kg^−1^, 117.1–149 mg Fe kg^−1^, 29.8–36.9 mg Zn kg^−1^, and 36.3–42.9 mg Mn kg^−1^, respectively.

## Discussion

We realized that the soil pH in the LYG orchards was lower than that in the HYG, resulting in reduced soil P_avail_ and exchangeable Ca concentrations (Table 2). Many studies have reported that low soil pH is considered the main causing affecting soil available P [40, 41]. After being fixed by Fe and Al, becomes transforms into an insoluble P compound, which reduces P-use efficiency and P uptake in plants [42]. The results from the DRIS indices also indicated that P was most deficient in the leaves at all pomelo growth stages (Table 4). The P index in the flowering (−24.0), fruit development (−15.6), and postharvest (−14.3) stages showed a negative value. In this study, we did not examine the Fe and Al content in the soils of pomelo orchards. However, our previous study [43] indicated that Al and Fe concentrations in the surface soil (0–20 cm) were 5.36 meq Al^3+^ 100g^−1^ and 5.20 g Fe^2+^ kg^−1^, respectively. Therefore, decreasing the concentration of these elements is necessary to improve soil available P in pomelo orchards. Considering the results of our previous studies in fruit orchards [4, 5], we recommend that gardeners use lime and soil amendment (compost or biochar) applications for their orchards. This is because they would increase soil pH and exchangeable Ca and decrease the contents of Al and Fe, thus improving the contents of soil available P and exchangeable cations. Previous studies [44, 45] have reported that a decrease in exchangeable Fe and Al cation concentrations increases soil P_tot_ and P_avail_ concentrations and inorganic and organic P fractions.

We successfully established the DRIS norms and indices for pomelos (Tables 3 and 4). The nutrient index was determined using the DRIS norms with high reliability, as they explained the relationship between the DRIS index and its concentration in leaves by a regression equation (Fig 3). These results were consistent with those of Teixeira et al. [46], Aliyu et al. [35], and Morales et al. [29]. They indicated that the DRIS method was more accurate and reliable for diagnosing the nutritional status of plants when there was a strong positive correlation between DRIS indices and their concentrations in leaves. We observed that N, P, and Fe were limited to the leaves at the flowering stage (Table 4). Meanwhile, the nutrients P, K, Fe, and Mn were limited at the fruit development stage, and N and P were deficient at the stage postharvest. Therefore, macronutrients such as N, P, and K were the most deficient in the leaves during the pomelo growth stages. As explained above, high soil acidity (pH < 5) is a reason for the decreased P concentration in pomelo leaves. According to Li et al. [47] low soil pH negatively affected P concentration in pummelo leaves. Therefore, improving soil pH is the most optimal solution for decreasing P deficiency in pomelo leaves.

An imbalanced chemical fertilizer supply affects leaf nutrient concentrations [48, 49]. Our previous study indicated that growers in the region applied less than the recommended amounts of N−P−K [37]. The means of N−P−K fertilizers applied by the farmers were 652−164−149 g tree^−1^ year^−1^, respectively, while the Southern Horticultural Research Institute (SOFRI)-Vietnam recommended 900 N, 600 P, and 850 K (g tree^−1^ year^−1^), respectively. This may lead to insufficient nutrients for pomelo growth and development, decreasing their concentrations in leaves. Various studies have reported that plants received lower than the required nutrient amounts, leading to decreased nutrient concentrations in leaves, which negatively affected the growth and productivity of crops [49–51]. Therefore, we suggest that farmers should apply N−P−K fertilizers based on the SOFRI recommendation. The results showed that most pomelo orchards were not deficient in Ca, Mg, Cu, Zn, or Mn at any of the three growth stages (Table 4). As mentioned in the methods, farmers only applied N−P−K compound fertilizers, but these contained trace elements. However, we could not determine the exact dose of trace elements applied to pomelo orchards. However, this may have led to improved concentrations of these elements in the soil. Therefore, pomelo trees can use/uptake nutrients during their growth and developmental stages. In cases of Ca and Mg, gardeners did not apply any fertilizers that contained Ca or Mg via the roots. Nevertheless, these were supplied through foliar fertilization during the fruit development stage to prevent fruit cracking, resulting in increased concentrations of Ca and Mg in pomelo leaves. The foliar application of nutrients is a strategy to supplement nutrients for crops because it is not affected by soil pH or root health [36, 52]. Hence, we did not observe shortages in Ca and Mg contents in pomelo leaves.

In this study, we observed that the concentrations of mineral nutrients in pomelo leaves were highest in the post-harvest stage (Table 5). This may be because the pomelo trees do not produce flowers or fruits at this stage. Therefore, most of the nutrients are concentrated in the leaves. Nutrients in leaves are used to synthesize soluble compounds that play an important role in the formation of flowers and improve the fruit-set ratio during the flowering stage. Similarly, nutrients in leaves improve fruit size and quality during fruit development. These results agree with the findings of Raese et al. [53] and Lin et al. [54]. We proposed optimal nutrient ranges for pomelos at different growth stages (Table 6). These standard concentrations agree with the results of Nguyen et al. [55], who reported that the optimal nutrient (N, K, Ca, and Zn) concentrations in pomelo leaves in Thailand were 26.2–28.5 g kg^−1^, 17.9–22.1 g kg^−1^, 25.4–42.0 g kg^−1^, and 33.1–35.0 mg kg^−1^, respectively. Another study in China showed that the mean nutrient concentrations in pomelo leaves were 25.1 g N kg^−1^, 1.42 g P kg^−1^, 15.4 g K kg^−1^, 31.1 g Ca kg^−1^, 44.9 g Cu kg^−1^, 35.0 g Zn kg^−1^, and 78.0 g Mn kg^−1^ [47]. Therefore, we recommend that growers use this standard concentration for pomelo cultivation and production. These results will help determine pomelo health and nutrient demands at different growth stages. Therefore, farmers could apply suitable amounts of fertilizer to their orchards, resulting in improved incomes and benefits.

## Conclusions

The pH of the soil in pomelo orchards in the VMD was low, leading to a decrease in soil available P. The DRIS indices showed that the concentrations of macronutrients (N, P, and K) in pomelo leaves were the most deficient compared with those of micronutrients during the different growth stages. The micronutrient (Cu, Fe, Zn, and Mn) indices explained the leaf nutrient content more clearly than the macronutrient indices. Therefore, improving soil acidity is considered the best method for enhancing leaf nutrient concentrations. Moreover, farmers need to apply enough amounts of N−P−K for pomelo based on the recommendation of fruit research institutes to improve their contents in leaves.
