# Correction: Determination of nutritional sufficiency ranges for pomelo (*Citrus grandis* Osbeck) grown on alluvial soils using DRIS

**DOI:** 10.1371/journal.pone.0339695

**Published:** 2025-12-23

**Authors:** Nguyen Kim Quyen, Le Van Dang, Ngo Phuong Ngoc, Pham Thi Phuong Thao, Ngo Ngoc Hung

There are errors in the author affiliations. The correct affiliations are as follows:

Nguyen Kim Quyen ^1^, Le Van Dang ^2,3^, Ngo Phuong Ngoc ^2^, Pham Thi Phuong Thao ^2^, Ngo Ngoc Hung ^2^

**1** Faculty of Agriculture and Fishery, University of Cuu Long, Vinh Long Province, Vietnam, **2** College of Agriculture, Can Tho University, Can Tho City, Vietnam, **3** United Graduate School of Agricultural Science, Tokyo University of Agriculture and Technology, Tokyo, Japan.
